# Computed Tomography Following Body Stuffing Heroin

**DOI:** 10.5811/westjem.2015.8.27431

**Published:** 2015-12-01

**Authors:** Sean P. Nordt, Marissa Camilon

**Affiliations:** * USC Keck School of Medicine, Section of Toxicology, Department of Emergency Medicine, Los Angeles, California; † LAC+USC, Department of Emergency Medicine, Los Angeles, California

## CASE

**Figure 1:**
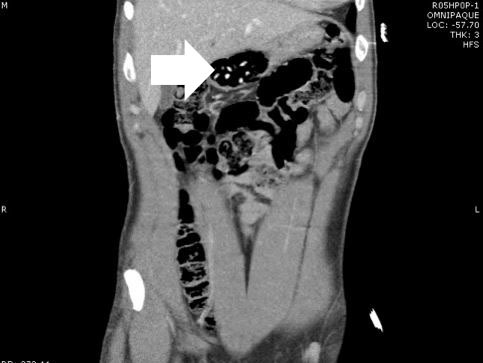
Coronal view of abdomen. Arrow denoting multiple drug packets in distal stomach.

**Figure 2:**
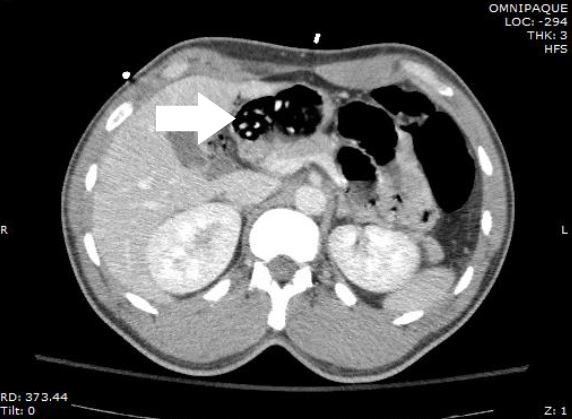
Axial view identifying multiple drug packets (arrow).

A 37-year-old male presented to the emergency department (ED) in police custody for “medical clearance” before being taken to jail. The patient was approached by police officers for suspicion of selling illicit drugs. When approached by police he ran away and was witnessed to swallow several small plastic baggies suspected to contain heroin. He was apprehended and brought to the ED. On arrival, he was asymptomatic with a blood pressure 144/83mmHg, heart rate 67bpm, respiratory rate of 19bpm, oxygen saturation of 99% on room air and afebrile. A Glasgow coma score was 15 and he was alert and oriented to person, place and time. Patient had a negative review of systems. On physical examination pupils were 4mm and reactive to light, lungs clear to auscultation and had normal respiratory rate with normal cardiovascular exam. Abdomen was soft, non-tender and non-distended with present bowel sounds. The patient admitted to ingesting approximately 20 packets of heroin to avoid being charged with possession. The patient declined activated charcoal and whole bowel irrigation (WBI) with polyethylene glycol-electrolyte solution (PEG-ELS). The patient declined a urine toxicology immunoassay screen. A computed tomography (CT) of his abdomen with contrast was obtained and read as normal except for a cluster of foreign bodies within the distal stomach likely contained within a plastic bag (Figures 1 and 2).

## DISCUSSION

Ingesting illicit substances generally falls into two broad categories: “body packing” where illicit substances are deliberately ingested as a means for transporting illicit drugs, and “body stuffing.”^1^,^2^ Body stuffing as in case presented is hastily ingesting drugs as means of evading possession charges from law enforcement. The major differences between body packing and body stuffing are the amount ingested, which is usually a large amount with body packing and also the wrapping of the illicit substance itself. With body packing the illicit drug is usually well wrapped often with double layers of condoms or balloons to prevent inadvertent rupture of the packets.^1^,^2^ As body stuffing is not usually pre-planned, the packets are often poorly wrapped and contained in the plastic baggies measured out in the amount by which they are generally sold. As such, body stuffers can be at increased risk for acute poisonings compared to body packers. Body packers may present with bowel obstruction. 1 However, due to the large amount of drug contained in body packers, if they do rupture it can be fatal, particularly with cocaine or methamphetamine.^1^ The patient presented ingested heroin by history. Heroin is somewhat easier to manage; if the patient had developed respiratory depression the opioid-specific reversal agent naloxone would have been administered, including continuous naloxone infusion.^1^ Plain abdominal radiographs are often negative following body stuffing and even packing, and as such a negative radiograph cannot exclude ingestion particularly with smaller number of packets ingested.^1^,^2^ Multi-detector CTs are much better at detecting drug packets than conventional radiology with a reported sensitivity and specificity of 95 to 100% but may not detect all packets.^2^,^3^ Consideration of CT abdominal imaging can be considered following plain abdominal radiographs, particularly if negative and high clinical suspicion. Anticipation of clinical signs of toxicity should be monitored for, as there may be a delay in the onset of toxicity from late opening of ingested packets. The role of WBI with PEG-ELS is better described with body packers than body stuffers but was considered in this case based on the large amount of packets ingested.^1^,^3^ Activated charcoal also may have a role with the goal of binding up leaking contents of packets before they reach systemic circulation.^1^ In patients who remain asymptomatic the general recommendation is to allow spontaneous passage.^1^ He was admitted to a monitored setting and observed until the packets were passed in the stool. The patient remained asymptomatic throughout his hospital course and was discharged to jail.
